# Association of rs440446 in the APOE intron region with multidimensional cognitive function, lipid/metabolic markers, and spontaneous neural activity in Chinese urban community-dwelling older adults

**DOI:** 10.3389/fnins.2025.1629254

**Published:** 2025-07-16

**Authors:** Zhiyuan Wang, Hongyu Guo, Jianshuai Li, Xuejie Hu, Xin Li, Jinping Sun

**Affiliations:** ^1^Department of Emergency Medicine, The Affiliated Hospital of Qingdao University, Qingdao, China; ^2^Health Management Center, The Affiliated Hospital of Qingdao University, Qingdao, China; ^3^State Key Laboratory of Cognitive Neuroscience and Learning, Beijing Normal University, Beijing, China

**Keywords:** rs440446, APOE, multi-dimensional cognitive function, resting-state functional magnetic resonance, blood lipids and metabolic markers

## Abstract

**Background:**

rs440446 (+ 113G/C), located in the intron of the APOE gene, has unclear effects on cognition, blood lipids, metabolic markers, and neural activity in older Chinese adults.

**Methods:**

A total of 505 older adults from communities in Qingdao were enrolled and categorized into C-allele carriers (*n* = 451) and GG homozygotes (*n* = 54) based on the sequencing result. Neuropsychological tests, lipid/metabolic markers (*n* = 326), and resting-state fMRI (rs-fMRI) data (*n* = 216) were collected. Multivariable linear regression was used to assess associations between rs440446 and cognitive performance, lipids, and metabolic indicators. Subgroup analyses stratified by age, gender, and APOE ε4 status were conducted, along with interaction tests. Propensity score matching (PSM) was applied to balance sample sizes in the rs-fMRI subgroup. Fractional amplitude of low-frequency fluctuations (fALFF) was used to evaluate differences in spontaneous neural activity, and Pearson correlations were calculated between fALFF values and cognitive function.

**Results:**

rs440446 GG homozygotes were associated with poorer language function. This association was consistently observed in female, ≥ 60 years old, and APOE ε4 non-carrier subgroups. However, no significant relationship was found between the GG genotype and lipid or metabolic markers. In fALFF analysis, GG homozygotes exhibited decreased spontaneous neural activity in the left middle occipital gyrus (MOG.L) and right cuneus (CUN.R), while showing increased activity in the right superior temporal gyrus (STG.R) and left fusiform gyrus (FFG.L). Additionally, fALFF values in the CUN.R were positively correlated with attention, whereas fALFF values in the STG.R were negatively correlated with memory.

**Conclusion:**

rs440446 GG homozygote may be associated with alterations in language function and spontaneous neural activity among older adults.

## Introduction

The rapid aging of the global population is pushing cognitive disorders toward a public health crisis. China, as one of the fastest-aging countries, has an Alzheimer’s disease (AD) prevalence rate of 3.9% among people over 60 (Jia et al., 2020). In the US, AD ranks as the fifth leading cause of death for those aged 65 and older (Alzheimer’s and Dementia, 2024). This neurodegenerative disease leads to personal disability, high caregiving costs for families, and reduced social productivity, making it a major challenge for healthy aging worldwide (Alzheimer’s and Dementia, 2024). Notably, the 2021 Global Burden of Diseases Study revealed that China bears the heaviest AD-related disease burden among G20 nations (Yang et al., 2024), highlighting the urgent need to study its risk factors and potential interventions. Genetic factors play a central role in AD pathogenesis, accounting for approximately 70% of the attributable risk (Silva et al., 2019). The Apolipoprotein E (APOE) gene, a key genetic regulator whose protein product influences AD through lipid transport, brain cell communication, and inflammation control, plays a crucial role in AD pathogenesis (Jackson et al., 2024; Raulin et al., 2022). The three main APOE alleles (ε2, ε3, ε4) are determined by two single nucleotide polymorphisms (SNPs): rs429358 (+ 334 T/C) and rs7412(+ 472 C/T) (Raulin et al., 2022). These combinations result in different risk profiles: ε2 is protective against AD (Martens et al., 2022; Raulin et al., 2022; Serrano-Pozo et al., 2021), ε3 is neutral, and ε4 represents the strongest genetic risk factor (Martens et al., 2022; Zhao et al., 2018). Recent studies show ε4 affects not only amyloid plaques but also tau pathology, neuroinflammation, and blood-brain barrier dysfunction (Martens et al., 2022; Serrano-Pozo et al., 2021; Zhao et al., 2018). However, the phenotypic expression of APOE alleles may be dynamically regulated by other genetic variants (such as linkage disequilibrium patterns) or environmental factors (Choi et al., 2019; Kulminski et al., 2020), suggesting that greater attention should also be paid to the non-coding regions of the APOE gene.

APOE rs440446 (+ 113 G/C) is located in the intron region (Komurcu-Bayrak et al., 2011). Although it does not directly participate in mature mRNA coding, it may influence APOE function by regulating transcriptional efficiency or allele-specific splicing. Unlike the APOE promoter SNP rs405509 (-219 G/T), where the TT genotype is significantly associated with AD risk and abnormal resting brain activity (Rantalainen et al., 2019; Wu et al., 2021), the association between rs440446 and cognitive function remains controversial. While several studies have not identified a significant association between rs440446 and total Mini-Mental State Examination (MMSE) scores (Prada et al., 2014), longitudinal data indicate that carriers of the rs440446 C allele exhibit an upward trend in visuospatial abilities with increasing age; in contrast, GG homozygotes show a significant decline (Rantalainen et al., 2016). These findings suggest that the rs440446 polymorphism may influence the pathogenesis of AD through heterogeneous effects across multiple cognitive domains.

For instance, the interaction between rs440446 and lipid metabolism has been shown to be particularly pronounced in specific populations, such as those with high red meat consumption (Andreotti et al., 2009). A study based on multiple European cohort datasets found that among men carrying the APOE ε2/ε3 heterozygous genotype, those with both the APOE ε2/ε3 genotype and the rs440446 heterozygous (CG) variant exhibited significantly enhanced protection against AD compared to those carrying the rs440446 GG variant (Kulminski et al., 2021). In contrast, a study using the Helsinki Birth Cohort found no association between the number of C alleles at the rs440446 locus and dementia risk (Rantalainen et al., 2019), further highlighting that the impact of rs440446 on cognitive function may occur via complex gene–environment or gene–gene interactions. It is noteworthy that current research largely relies on tools such as the MMSE, which provide limited assessment across multiple cognitive domains, including memory, executive, and language function. Therefore, future studies should aim for more comprehensive evaluations of rs440446’s effects on distinct cognitive domains (Del Bene et al., 2023). Moreover, the potential neuroimaging mechanisms underlying rs440446-related cognitive changes—such as its influence on spontaneous neural activity—remain insufficiently explored. Further investigation into these mechanisms may not only elucidate how rs440446 affects cognition but also provide critical insights for the development of novel preventive and therapeutic strategies.

Although the role of APOE gene polymorphism has been extensively studied, the functional significance of the intronic variant rs440446 remains poorly understood. Limited evidence exists regarding its association with cognitive function, particularly in elderly Chinese populations. Moreover, it remains unclear whether rs440446 influences cognitive performance across multiple domains or how its polymorphism relates to lipid metabolism and spontaneous neural activity. Building on this knowledge gap, we hypothesize that the APOE rs440446 polymorphism affects performance across multiple cognitive domains in Chinese older adults and plays a role in lipid metabolism regulation. Additionally, it may be linked to changes in resting-state neural activity. Given that APOE ε4, age, and gender are well-established factors influencing cognitive function, we further aim to investigate whether these variables moderate the relationship between rs440446 and cognitive outcomes—an aspect that remains underexplored. The results of this study are expected to contribute novel insights into the regulatory mechanisms of SNPs located in the intronic region of the APOE gene, offering translational neuroscience evidence for their potential role in cognitive aging.

## Materials and methods

### Participants

**FIGURE 1:**
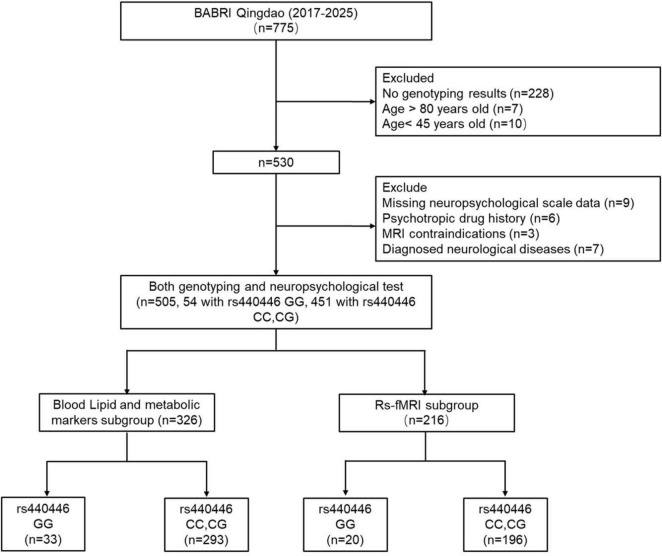
A detailed flowchart of participant recruitment and subgroup division. BABRI, Beijing Aging Brain Rejuvenation Initiative.

This cross-sectional study recruited 775 community-dwelling participants from urban areas of Qingdao, China, as part of the Beijing Aging Brain Rejuvenation Initiative (BABRI) cohort, a community-based longitudinal study launched in 2008 to investigate preclinical stages of dementia, develop population-level cognitive impairment prevention strategies, and establish a platform for scientific research and clinical trials (Yang et al., 2021). Inclusion/Exclusion Criteria: (1) lack of genotyping data (*n* = 228); (2) age outside the 45–80 years range (*n* = 17); (3) missing neuropsychological data (*n* = 9); (4) history of psychotropic medication use or diagnosed psychiatric disorders (*n* = 6); (5) MRI contraindications (*n* = 3); and (6) diagnosed neurological diseases (*n* = 7). Following these exclusions, 505 participants were included in the final analysis. This cohort is further divided into two subgroups according to the completeness of the corresponding data: a blood lipid and metabolic biomarker subgroup (*n* = 326) comprising participants with successful biochemical testing results and an rs-fMRI subgroup (*n* = 216) comprising individuals who underwent rs-fMRI scans. The study protocol was approved by the Ethics Committee of the Affiliated Hospital of Qingdao University (Approval No.: ICBIR_A_0041_002_02). Written informed consent was obtained from all participants before data collection (see Figure 1 for details).

### Demographic and clinical characteristics

Demographic data (age, gender, ethnicity, education level, height, weight, smoking status, and alcohol consumption) were collected through standardized questionnaires and face-to-face interviews. Since all participants were of Han Chinese ethnicity, this variable was excluded from subsequent statistical analyses. Body mass index (BMI) was calculated as weight in kilograms divided by the square of height in meters (kg/m^2^). Hypertension was defined as self-reported physician diagnosis, current or prior use of antihypertensive medications, or measured systolic/diastolic blood pressure ≥ 140/90 mmHg. Diabetes was diagnosed based on self-reported history, current use of insulin or oral hypoglycemic agents, or HbA1c ≥ 6.5%. Cerebrovascular disease was assessed by self-reported history of stroke or radiologically confirmed small vessel disease on MRI. Cardiovascular disease was defined as a self-reported history of coronary artery disease, heart failure, revascularization procedures (e.g., stent placement), or current use of cardiovascular medications. Hyperlipidemia was determined by self-reported history, current lipid-lowering therapy, or abnormal lipid profiles. Smoking: defined as current smoking or a history of 100 or more cigarettes in a lifetime. Alcohol consumption: current or past intake of ≥ 1 standard drink/week for ≥ 6 months. All clinical assessments integrated self-reported data, medication records, and objective laboratory and imaging findings.

### Neuropsychological assessment

This study utilized a battery of neuropsychological scales to comprehensively assess multi-dimensional cognitive function in all participants. The MMSE, a widely used scale, was employed to evaluate general cognitive function; its specific assessment procedures were not to be described in detail. The Auditory Verbal Learning Test (AVLT) consists of five components: N1–N3 assess working memory through immediate recall trials of 12 Chinese words read by the examiner; N4 evaluates short-delay recall after a 5-min interval; and N5 tests long-delay recall following a 20-min delay. For N4 and N5, participants recalled words without re-exposure. The copy portion of the Rey-Osterrieth Complex Figure (ROCF) test was used to assess visuospatial abilities, while its recall portion evaluated visual memory. In ROCF-copy, participants were asked to draw a complex figure using a red pen for the first four strokes, followed by a black pen. For ROCF-recall, participants reproduced the figure from memory after 20 min. The Clock Drawing Test (CDT) also assessed visuospatial abilities. Participants drew a clock with hands set to 1:50, using a red pen for six initial strokes followed by a black pen. The Stroop Color and Word Test (SCWT) was used to evaluate the individual’s color word naming ability in Part A, color discrimination ability in Part B, and the ability to suppress cognitive interference in Part C. Part A: Reading color words (“red,” “yellow,” “blue,” “green”) randomly arranged. Completion time [SCWT-A (time)] and accuracy [SCWT-A (right)] were recorded. Part B: Identifying colors of patches. Time [SCWT-B (time)] and accuracy [SCWT-B (right)] were recorded. Part C: Naming font colors conflicting with word meanings. Time [SCWT-C (time)] and accuracy [SCWT-C (correct)] were recorded. For the Symbol Digit Modalities Test (SDMT), participants matched symbols to numbers within 90 s, measuring processing speed and attention. The Trail Making Test (TMT) comprises TMT-A, which is used to evaluate visual search ability, and TMT-B, which assesses executive function. TMT-A: Connecting numbers 1–25 sequentially; shorter times indicated better performance. TMT-B: Alternating between numbered squares and circles under time constraints. The completion time of TMT-A and TMT-B is the final data. The Verbal Fluency Test (VFT) included three subtests (animal/vegetable/fruit) requiring non-repeated item naming within 1 min each, assessing language function. The Boston Naming Test (BNT) evaluated visual naming ability and language function by identifying 30 pictured objects. All assessments used validated Mandarin versions with established cross-cultural reliability.

### Genotyping procedures

DNA extraction from peripheral blood samples of 530 participants was performed using standard protocols.

APOE genotyping was conducted using PCR-based methods (Applied Biosystems, Foster City, CA, United States). APOE genotypes were classified based on two single nucleotide polymorphisms (SNPs), rs429358 (+ 334 T/C) and rs7412 (+ 472 C/T), to identify APOE ε4 carriers (ε4 +) and non-carriers (ε4-). Among the 530 successfully genotyped participants, 95 were classified as APOE ε4 carriers (including ε2/ε4: 5, ε3/ε4: 81, ε4/ε4: 9), while 435 as APOE ε4 non-carriers (including ε2/ε2: 5, ε2/ε3: 64, ε3/ε3: 366). Additionally, all subjects underwent genotype analysis for rs440446 using a custom TaqMan SNP Genotyping Assay (Applied Biosystems, Foster City, CA, United States). Based on rs440446 genotypes, participants were stratified into C allele carriers (including CC and CG genotypes) and GG homozygotes. Initial genotyping identified 473 C allele carriers (CC: 223, CG: 250) and 57 GG homozygotes. Following additional quality control steps and exclusion criteria, a total of 505 subjects with complete genotype data were included in the final analysis (C allele carriers: 451, GG homozygotes: 54).

### Lipid and metabolic marker testing

This study collected venous blood samples from participants’ antecubital veins between 7 and 9 AM after ≥ 8 h of fasting. Lipid Profile Analysis was performed using an automated biochemical analyzer employing enzymatic colorimetric assays for High-Density Lipoprotein (HDL), Low-Density Lipoprotein (LDL), Total Cholesterol (TC), and Triglycerides (TG). For Metabolic Marker Detection, Hemoglobin A1c (HbA1c) levels were determined by High-Performance Liquid Chromatography (HPLC) due to its high precision, while homocysteine (HCY) levels were measured in fasting samples using fluorescence polarization immunoassay (FPIA). In the end, 326 subjects completed the lipid and metabolic marker analyses. All laboratory tests were performed strictly according to the manufacturers’ guidelines by skilled technicians at the Department of Laboratory Medicine, Affiliated Hospital of Qingdao University.

### MRI acquisition

Resting-state functional MRI (rs-fMRI) and structural MRI scans were acquired for partial participants using a General Electric (GE) Signa HDX 3.0 Tesla scanner at the Affiliated Hospital of Qingdao University, China. Participants were instructed to remain awake, keep their eyes closed, and minimize head movement during scanning. Foam padding and noise-reducing headphones were utilized to mitigate motion artifacts and scanner noise. The rs-fMRI data were obtained via an echo-planar imaging (EPI) sequence with the following parameters: 33 axial slices, repetition time (TR) = 2,000 ms, echo time (TE) = 30 ms, slice thickness = 3.5 mm, flip angle = 90°, acquisition matrix = 64 × 64, field of view (FOV) = 200 × 200 mm, and 210 volumes. High-resolution T1-weighted images were acquired using a magnetization-prepared rapid gradient-echo (MPRAGE) sequence: 176 sagittal slices, TR = 1,900 ms, TE = 3.44 ms, isotropic voxel size = 1 × 1 × 1 mm^3^, acquisition matrix = 256 × 256, slice thickness = 1 mm, and FOV = 256 × 256 mm.

### Resting-state functional MRI data preprocessing

**FIGURE 2:**
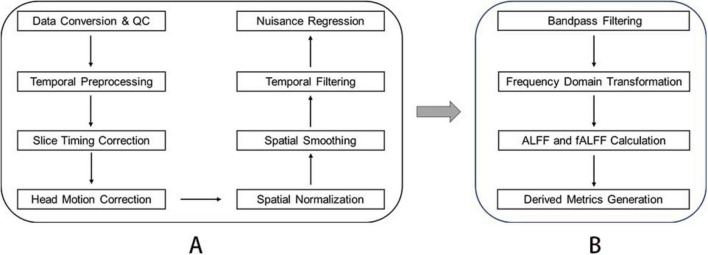
Data processing flow of resting-state functional magnetic resonance. **(A)** Fundamental steps in the preprocessing of resting-state functional MRI Data. **(B)** Key steps for deriving fALFF metrics.

All preprocessing steps were implemented using RESTplus v1.31^1^ based on SPM12^2^ within MATLAB 2013b (MathWorks) with the following workflow: (1) Data Conversion and Quality Control, Raw DICOM files were manually converted to NIFTI format using dcm2nii,^3^ followed by visual inspection to exclude volumes with acquisition artifacts or missing slices. (2) Temporal Preprocessing, the first 10 volumes were discarded to account for magnetic field stabilization and scanner equilibrium. (3) Slice timing correction was applied to the remaining 200 volumes using ascending interleaved acquisition order (reference slice: middle temporal slice). (4) Head Motion Correction and realignment parameters were estimated via rigid-body transformation (6 degrees of freedom). Participants exceeding frame-wise displacement (FD) thresholds of 2.5 mm translation or 2.5° rotation were excluded from subsequent analyses. (5) Spatial Normalization, T1-weighted images were segmented and non-linearly registered to the Montreal Neurological Institute (MNI) ICBM152 template using the DARTEL algorithm in SPM12. Normalized functional images were resampled to isotropic 3 mm^3^ voxels. (6) Spatial Smoothing, a Gaussian kernel with 6 mm full-width at half-maximum (FWHM), was applied to enhance signal-to-noise ratio. (7) Temporal Filtering and linear trends were removed to mitigate scanner drift effects. (8) Nuisance Regression, spurious variances were regressed using covariates including white matter signals, cerebrospinal fluid signals, and six head motion parameters. The brief preprocessing workflow is illustrated in Figure 2A.

### Fractional amplitude of low-frequency fluctuations analysis

Bandpass filters (0.01–0.08 Hz) were applied to eliminate the effects of low-frequency drift and high-frequency noise from the time series data. The Fast Fourier Transform (FFT) was then used to convert the filtered time series into the frequency domain, thereby obtaining the power spectrum. For each voxel, low-frequency fluctuations (ALFF) amplitude was calculated as the square root of the average power within the 0.01–0.08 Hz range. The fractional amplitude of low-frequency fluctuations (fALFF) was computed as the ratio of the root-mean-square (RMS) amplitude in the low-frequency band (0.01–0.08 Hz) to the RMS amplitude across the entire frequency range (0–0.25 Hz). In addition to the raw fALFF values, two processed derivative measures were obtained during the analysis. The first is mfALFF, calculated by normalizing each voxel’s fALFF value by the participant-specific global mean fALFF, resulting in an individual-level standardized measure. The second is zALFF, computed by subtracting the global mean fALFF from each voxel’s fALFF value and then dividing by the global standard deviation, yielding a standardized z-score. Given its stability and interpretability in group comparisons, zfALFF was selected for subsequent statistical analyses. The essential steps are shown in Figure 2B.

### Statistical analysis

(1) Continuous variables are presented as mean ± standard deviation (SD), while categorical variables are expressed as frequency (percentage). Intergroup differences between the rs440446 C allele carrier group and GG homozygous group were assessed using independent two-sample *t*-tests for continuous variables and Chi-square (χ^2^) tests for categorical variables. (2) The neuropsychological tests with significant differences between the two groups were included in the multivariate linear regression model to evaluate the relationship between rs440446 (GG homozygotes vs. C allele carriers) and multi-dimensional cognitive function. Four models were constructed, Model 1: unadjusted (crude model); Model 2: adjusted for demographic variables (age, gender, education years); Model 3: Model 2 + APOE ε4 and BMI; Model 4: Model 3 + hypertension, diabetes, cardiovascular disease, cerebrovascular disease, hyperlipidemia, smoking, and alcohol use. (3) We conducted subgroup analyses and interaction tests based on gender (female vs. male), age (< 60 vs. ≥ 60 years), and APOE ε4 carrier status (APOE ε4 carriers vs. APOE ε4 non-carriers) to examine whether the association between rs440446 and cognitive function was modified by these factors. All subgroup models were adjusted for the same covariates as those included in Model 3 of the main analysis, with the exception of the stratification variable itself. (4) Lipid subgroup analysis: data presentation and intergroup comparisons followed the abovementioned methods. The relationship between rs440446 and blood lipid profile was analyzed by multivariate linear regression models. It is worth noting that in Model 4, hyperlipidemia was excluded when the relationship between rs440446 and lipids (LDL, HDL, TC, TG) was analyzed, and diabetes was excluded when the relationship between rs440446 and HbA1c was analyzed. (5) Rs-fMRI subgroup analysis: Propensity score matching (PSM) was performed to address the problem of significant differences in sample sizes between the GG homozygous group (*n* = 20) and C allele carrier group (*n* = 196). PSM was performed using age, sex, education, and BMI as matching variables. The 1:1 nearest neighbor matching algorithm is adopted, and the matching tolerance is 0.02. (6) Comparison of fALFF: fALFF differences between groups were analyzed using general linear models (GLMs) in SPM12. The covariates were adjusted for years of age, gender, education, APOE ε4 status and BMI. The multiple comparison correction of fALFF analysis was performed using Cluster-level FDR (voxel-level *p* < 0.001). (7) Brain function-cognition correlation analysis: Brain regions showing significant fALFF differences between groups were defined as regions of interest (ROIs). The fALFF values from these ROIs were extracted, and their correlations with neuropsychological assessment scores were evaluated using Pearson’s correlation analysis. A significance level of < 0.05 (two-sided *p*-value) was used to determine statistical significance. Data processing and analysis were performed using R version 4.4.3 and Zstats v1.0.^4^ The PSM was done based on IBM SPSS Statistics 29.^5^

## Results

### Demographic and baseline characteristics

This cross-sectional study eventually included 505 community older adults from urban areas of Qingdao, who were divided into two genotypic groups: 451 (89.31%) C allele carriers and 54 (10.69%) GG homozygotes. Comparative analysis of baseline characteristics revealed comparable profiles between groups, as shown in Table 1. Age distributions showed no significant difference (C carriers: 61.34 ± 8.16 years vs. GG homozygotes: 63.37 ± 9.04 years, *p* = 0.089), nor did gender composition (female: 61.64 vs. 61.11%, *p* = 0.94). There was no significant difference in years of education (10.77 ± 3.48 vs. 10.14 ± 3.38, *p* = 0.209). BMI (kg/m^2^) (24.70 ± 3.79 vs. 23.78 ± 3.59, *p* = 0.057) showed comparable values approaching but not reaching statistical significance. No between-group disparities emerged in clinical comorbidities, including hypertension, diabetes, cardiovascular disease, cerebrovascular disease, and hyperlipidemia, nor lifestyle factors such as smoking and alcohol consumption (all *p* > 0.05). However, a marked genotypic divergence was observed in APOE ε4 status, with the GG homozygotes exhibiting substantially higher carrier rates (48.10 vs. 13.75%, *p* < 0.001). Subsequent analyses adjusted for APOE ε4 status to account for this genetic confounding.

**TABLE 1:** Baseline characteristics of between the rs440446 C allele carriers and GG homozygotes.

Variables	Overall (*n* = 505)	CC, CG (*n* = 451)	GG (*n* = 54)	Statistics	*p*-value
Age (years)	61.56 ± 8.27	61.34 ± 8.16	63.37 ± 9.04	*t* = -1.71	0.089
Education (years)	10.70 ± 3.47	10.77 ± 3.48	10.14 ± 3.38	*t* = 1.26	0.209
BMI (kg/m^2^)	24.61 ± 3.77	24.70 ± 3.79	23.78 ± 3.59	*t* = 1.70	0.089
Gender, *n* (%)				χ^2^ = 0.01	0.940
Female	311 (61.58%)	278 (61.64%)	33 (61.11%)		
Male	194 (38.42%)	173 (38.36%)	21 (38.89%)		
APOE ε4 carrier, *n* (%)				χ^2^ = 39.66	<0.001***
No	417 (82.57%)	389 (86.25%)	28 (51.90%)		
Yes	88 (17.43%)	62 (13.75%)	26 (48.10%)		
Hypertension, *n* (%)				χ^2^ = 2.31	0.128
No	317 (73.47%)	278 (61.64%)	39 (72.22%)		
Yes	188 (26.53%)	173 (38.36%)	15 (27.78%)		
Diabetes, *n* (%)				χ^2^ = 0.20	0.658
No	431 (85.35%)	386 (85.59%)	45 (83.33%)		
Yes	74 (14.65%)	65 (14.41%)	9 (16.67%)		
Cerebrovascular disease, *n* (%)				χ^2^ = 0.28	0.599
No	424 (83.96%)	380 (84.26%)	44 (81.48%)		
Yes	81 (16.04%)	71 (15.74%)	10 (18.52%)		
Cardiovascular disease, *n* (%)				χ^2^ = 2.13	0.145
No	427 (84.55%)	385 (85.37%)	42 (77.78%)		
Yes	78 (15.45%)	66 (14.63%)	12 (22.22%)		
Hyperlipidemia, *n* (%)				χ^2^ = 0.16	0.690
No	372 (73.66%)	331 (73.39%)	41 (75.93%)		
Yes	133 (26.34%)	120 (26.61%)	13 (24.07%)		
Smoking, *n* (%)				χ^2^ = 2.98	0.085
No	393 (77.82%)	346 (76.72%)	47 (87.04%)		
Yes	112 (22.18%)	105 (23.38%)	7 (12.96%)		
Alcohol use, *n* (%)				χ^2^ = 1.44	0.231
No	388 (76.83%)	343 (76.05%)	45 (83.33%)		
Yes	117 (23.17%)	108 (23.95%)	9 (16.67%)		

Continuous variables are presented as mean ± standard deviation (SD), while categorical variables are expressed as frequency (percentage). Intergroup differences between the rs440446 C allele carrier group and GG homozygous group were assessed using independent two-sample *t*-tests for continuous variables and Chi-square (χ^2^) tests for categorical variables. Statistical significance was defined as a two-tailed *p* < 0.05. BMI, body mass index; APOE, Apolipoprotein E. ***p < 0.001.

### The differences in multi-dimensional cognitive performance between GG homozygotes and C allele carriers

Significant disparities in cognitive function were observed between GG homozygotes and C allele carriers, with the former group demonstrating inferior performance across multiple neuropsychological domains, as shown in Table 2. GG homozygotes exhibited markedly lower scores on the MMSE (25.48 ± 3.98 vs. 26.88 ± 2.75, *p* = 0.015), indicating compromised global cognitive functioning. GG homozygotes exhibited pronounced deficits in memory-related tasks. Specifically, these individuals showed significantly lower performance in both immediate recall (AVLT-N1–N3: 14.61 ± 6.09 vs. 16.41 ± 5.51, *p* = 0.025) and long-delayed recall (AVLT-N5: 3.96 ± 3.16 vs. 4.80 ± 2.86, *p* = 0.045). A significant between-group difference was also found in total AVLT scores (22.81 ± 11.53 vs. 26.42 ± 10.52, *p* = 0.019). Although the difference in short-delayed recall (AVLT-N4: 4.44 ± 3.06 vs. 5.22 ± 2.80, *p* = 0.059) did not reach statistical significance, the observed trend indicates that GG homozygotes may have potential damage in this region. GG homozygotes exhibited impaired performance on SCWT Part B, with prolonged completion times (46.28 ± 22.07 vs. 41.64 ± 14.99 s, *p* = 0.043), indicating weaker color discrimination ability. Longer TMT-A completion times in GG homozygotes (70.56 ± 38.28 *vs*. 60.28 ± 27.29 s, *p* = 0.013), may reflect impaired visual search efficiency. GG homozygotes exhibited poorer SDMT performance (30.59 ± 13.18 vs. 35.15 ± 13.51, *p* = 0.019), reflecting information processing speed and attention deficits. Significant group differences emerged in vocabulary fluency tasks, with GG homozygotes generating fewer valid responses, VFT (36.67 ± 10.59 vs. 41.33 ± 9.35, *p* < 0.001), VFT-animal (14.24 ± 4.46 vs. 16.08 ± 4.31, *p* = 0.003), VFT-vegetable (12.06 ± 4.54 vs. 13.51 ± 3.91, *p* = 0.011), and VFT-fruit (10.37 ± 3.37 vs. 11.79 ± 3.21, *p* = 0.002). No statistically significant differences were detected between groups in SCWT-A (time, *p* = 0.076; right, *p* = 0.507), SCWT-B (right, *p* = 0.997), SCWT-C (time, *p* = 0.286; right, *p* = 0.072), ROCF-copy (*p* = 0.828), ROCF-recall (*p* = 0.074), CDT (*p* = 0.832), TMT-B (*p* = 0.248), and BNT (*p* = 0.161).

**TABLE 2:** Multidimensional cognitive function score difference between the rs440446 C allele carriers and GG homozygotes.

Variables	Overall (*n* = 505)	CC, CG (*n* = 451)	GG (*n* = 54)	Statistics	*p*-value
MMSE	26.73 ± 2.94	26.88 ± 2.75	25.48 ± 3.98	*t* = 2.50	0.015*
AVLT (N1–N3)	16.22 ± 5.59	16.41 ± 5.51	14.61 ± 6.09	*t* = 2.25	0.025
AVLT (N4)	5.13 ± 2.84	5.22 ± 2.80	4.44 ± 3.06	*t* = 1.89	0.059
AVLT (N5)	4.71 ± 2.90	4.80 ± 2.86	3.96 ± 3.16	*t* = 2.01	0.045*
AVLT (total)	26.04 ± 10.68	26.42 ± 10.52	22.81 ± 11.53	*t* = 2.36	0.019*
ROCF (copy)	32.55 ± 6.73	32.53 ± 6.76	32.74 ± 6.53	*t* = -0.22	0.828
ROCF (recall)	13.45 ± 8.16	13.67 ± 8.31	11.57 ± 6.55	*t* = 2.16	0.074
CDT	21.87 ± 6.05	21.85 ± 5.95	22.04 ± 6.90	*t* = -0.21	0.832
SCWT-A (time)	29.17 ± 9.24	28.92 ± 9.26	31.28 ± 8.94	*t* = -1.78	0.076
SCWT-A (right)	49.72 ± 1.29	49.71 ± 1.35	49.83 ± 0.61	*t* = -0.66	0.507
SCWT-B (time)	42.13 ± 15.94	41.64 ± 14.99	46.28 ± 22.07	*t* = -2.03	0.043*
SCWT-B (right)	49.08 ± 2.78	49.08 ± 2.87	49.07 ± 1.88	*t* = 0.00	0.997
SCWT-C (time)	88.85 ± 32.17	88.32 ± 31.76	93.26 ± 35.41	*t* = -1.07	0.286
SCWT-C (right)	46.03 ± 5.66	46.19 ± 5.52	44.72 ± 6.63	*t* = 1.80	0.072
SDMT	34.66 ± 13.54	35.15 ± 13.51	30.59 ± 13.18	*t* = 2.35	0.019*
TMT-A	61.38 ± 28.79	60.28 ± 27.29	70.56 ± 38.28	*t* = -2.49	0.013*
TMT-B	161.36 ± 69.57	160.12 ± 69.50	171.70 ± 69.97	*t* = -1.16	0.248
VFT	40.83 ± 9.59	41.33 ± 9.35	36.67 ± 10.59	*t* = 3.42	<0.001***
VFT-animal	15.89 ± 4.36	16.08 ± 4.31	14.24 ± 4.46	*t* = 2.96	0.003**
VFT-vegetable	13.36 ± 4.01	13.51 ± 3.91	12.06 ± 4.54	*t* = 2.54	0.011*
VFT-fruit	11.64 ± 3.25	11.79 ± 3.21	10.37 ± 3.37	*t* = 3.05	0.002**
BNT	22.71 ± 4.13	22.80 ± 4.08	21.96 ± 4.49	*t* = 1.40	0.161

MMSE, Mini-Mental State Examination; AVLT, Auditory Verbal Learning Test; ROCF, Rey-Osterrieth Complex Figure; CDT, Clock Drawing Test; SCWT, Stroop Color Word Test; SDMT, Symbol Digit Modalities Test; TMT, Trail Making Test; VFT, Verbal Fluency Test; BNT, Boston Naming Test. *p < 0.05; **p < 0.01; ***p < 0.001. Independent two-sample *t*-tests obtained the *p*-values.

### The relationship between rs440446 and multi-dimensional cognitive performance

Multivariate linear regression analyses with C allele carriers as the reference group revealed significant associations between GG homozygous carriers and cognitive decline across multiple domains, as shown in Table 3. In Model 1 (crude model), GG homozygotes showed significant correlations with all cognitive tests, but this result indicates a connection between this genotype and poorer cognitive performance. In Model 2 (adjusted for age, gender, education), GG homozygous carriers were negatively associated with MMSE [β = −1.12, 95% CI (−1.91, –0.34), *p* = 0.005], VFT [β = −3.59, 95% CI (−6.03, –1.14), *p* = 0.004], VFT-animal [β = −1.42, 95% CI (−2.57, –0.27), *p* = 0.016], VFT-vegetable [β = −1.05, 95% CI (−2.08, –0.01), *p* = 0.048], and VFT-fruit [β = −1.16, 95% CI (−2.02, –0.30), *p* = 0.009]. Further inclusion of BMI and APOE ε4 (Model 3) revealed that GG homozygous carriers were also negatively with MMSE [β = −0.83, 95% CI (−1.64, –0.01), *p* = 0.047] and part of the language function test, VFT [β = −3.47, 95% CI (−6.03, –0.91), *p* = 0.008], VFT-animal [β = −1.33, 95% CI (−2.54, –0.13), *p* = 0.030], and VFT-fruit [β = −1.1, 95% CI (−2.0, –0.2), *p* = 0.018]. Notably, the fully adjusted model (Model 4) revealed persistent negative effects exclusively in language function, VFT [β = −3.53, 95% CI (−6.07, –0.99), *p* = 0.007], VFT-animal [β = −1.34, 95% CI (−2.55, –0.13), *p* = 0.031], and VFT-fruit [β = −1.17, 95% CI (−2.06, –0.27), *p* = 0.011]. These results suggest that GG homozygotes may be associated with poorer language function and are not disturbed by other factors.

**TABLE 3:** The relationship between rs405509 and multidimensional cognitive function.

Variables	Reference	Model 1	Model 2	Model 3	Model 4
		β (95% CI) *p*-value	β (95% CI) *p*-value	β (95% CI) *p*-value	β (95% CI) *p*-value
MMSE	CC, CG	−1.40 (-2.22, –0.58) < 0.001***	−1.12 (−1.91, –0.34) 0.005**	−0.83 (−1.64, –0.01) 0.047*	−0.81 (−1.63, 0.01) 0.053
AVLT (N1–N3)	CC, CG	−1.80 (−3.38, –0.23) 0.025*	−1.07 (−2.43, 0.30) 0.127	−0.92 (−2.34, 0.51) 0.208	−0.99 (−2.42, 0.45) 0.180
AVLT (N5)	CC, CG	−0.84 (−1.65, –0.02) 0.045*	−0.48 (−1.20, 0.25) 0.197	−0.27 (−1.02, 0.48) 0.481	−0.29 (−1.05, 0.47) 0.462
AVLT (total)	CC, CG	−3.61 (−6.61, –0.60) 0.019*	−2.17 (−4.76, 0.43) 0.102	−1.60 (-4.30, 1.10) 0.246	−1.68 (−4.40, 1.04) 0.226
SCWT-B(time)	CC, CG	4.64 (0.16, 9.13) 0.043*	3.12 (−0.96, 7.20) 0.135	2.29 (−1.97, 6.56) 0.292	2.55 (−1.74, 6.83) 0.244
SDMT	CC, CG	−4.57 (−8.37, –0.76) 0.019*	−1.95 (−4.74, 0.85) 0.173	−0.91 (−3.82, 1.99) 0.537	−1.43 (−4.34, 1.49) 0.338
TMT-A	CC, CG	10.27 (2.19, 18.36) 0.013*	7.11 (−0.40, 14.61) 0.064	6.01 (−1.84, 13.86) 0.134	6.40 (−1.46, 14.27) 0.111
VFT	CC, CG	−4.67 (−7.34, –1.99) < 0.001***	−3.59 (−6.03, –1.14) 0.004**	−3.47 (−6.03 0.91) 0.008**	−3.53 (−6.07, –0.99) 0.007**
VFT-animal	CC, CG	−1.84 (−3.06, –0.62) 0.003**	−1.42 (−2.57, –0.27) 0.016*	−1.33 (−2.54, –0.13) 0.030*	−1.34 (−2.55, –0.13) 0.031*
VFT-vegetable	CC, CG	−1.46 (−2.58, –0.33) 0.011*	−1.05 (−2.08, –0.01) 0.048*	−1.07 (−2.15, 0.02) 0.054	−1.07 (−2.15, 0.02) 0.055
VFT-fruit	CC, CG	−1.42 (−2.33, –0.51) 0.002**	−1.16 (−2.02, –0.30) 0.009**	−1.10 (−2.00, –0.19) 0.018*	−1.17 (−2.06, –0.27) 0.011*

MMSE, Mini-Mental State Examination; AVLT, Auditory Verbal Learning Test; SCWT, Stroop Color Word Test; SDMT, Symbol Digit Modalities Test; TMT, Trail Making Test; VFT, Verbal Fluency Test; BNT, Boston Naming Test. Model 1: crude model; Model 2: adjusted for age, gender, and education; Model 3: Model 2 + APOE ε4 + BMI; Model 4: Model 3 + hypertension, diabetes, cardiovascular disease, cerebrovascular disease, hyperlipidemia, smoking, alcohol consumption. β, regression coefficient; 95% CI, 95% confidence interval. *p < 0.05; **p < 0.01; ***p < 0.001.

### Subgroup analysis of rs440446 by age, gender, and APOE ε4

**FIGURE 3:**
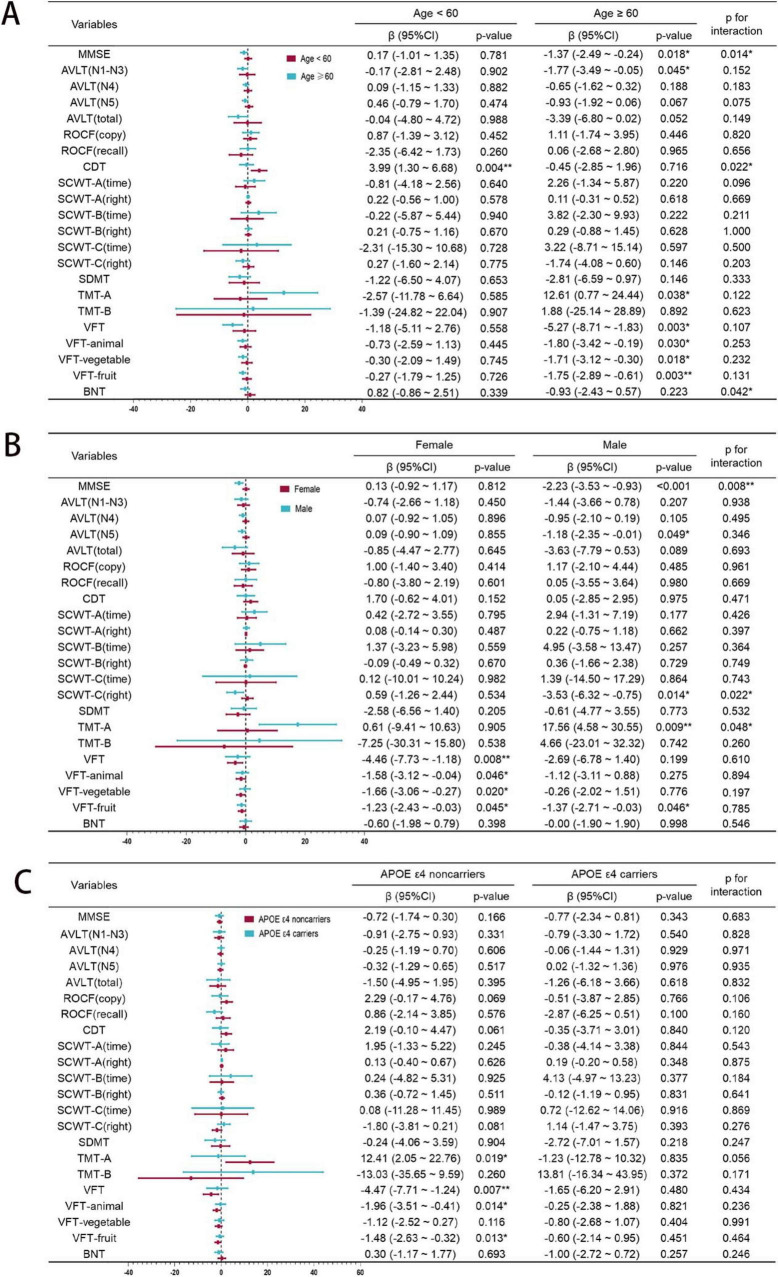
Subgroup analysis of rs440446 by age, gender, and APOE ε4. **(A)** Age subgroup. **(B)** Gender subgroup. **(C)** APOE ε4 subgroup. MMSE, Mini-Mental State Examination; AVLT, Auditory Verbal Learning Test; ROCF, Rey-Osterrieth Complex Figure; CDT, Clock Drawing Test; SCWT, Stroop Color Word Test; SDMT, Symbol Digit Modalities Test; TMT, Trail Making Test; VFT, Verbal Fluency Test; BNT, Boston Naming Test; 95% CI, Confidence Interval. *p < 0.05; **p < 0.01.

To explore the potential impacts of age, gender, and APOE ε4 carrying status on the relationship between rs440446 and multidimensional cognitive function, a subgroup analysis was conducted, with results illustrated in Figure 3. In the subgroup aged < 60 years, GG homozygotes were significantly positively correlated with CDT scores [β = 3.99, 95% CI (1.30, 6.68), *p* = 0.004]. However, in the subgroup aged ≥ 60 years, they showed an insignificant negative trend, and the interaction between rs440446 and age was statistically significant in CDT (p for interaction = 0.022). In the subgroup aged ≥ 60 years, GG homozygotes were significantly negatively correlated with MMSE [β = −1.37, 95% CI (−2.49, –0.24), *p* = 0.018], while in the subgroup aged < 60 years, they showed a non-significant positive trend. Meanwhile, the interaction between rs440446 and age was also statistically significant in MMSE (*p* for interaction = 0.014). Further analysis showed that in the subgroup aged ≥ 60 years, GG homozygotes were significantly correlated with multiple cognitive tests, including AVLT [N1–N3, β = −1.77, 95% CI (−3.49, −0.05)], VFT [β = −5.27, 95% CI (−8.71, –1.83), *p* = 0.003], VFT-animal [β = −1.80, 95% CI (−3.42, –0.19), *p* = 0.030], VFT-vegetable [β = −1.71, 95% CI (−3.12, –0.30), *p* = 0.018], VFT-fruit [β = −1.75, 95% CI (−2.89, –0.61), *p* = 0.003], and TMT-A [β = 12.61, 95% CI (0.77, 24.44), *p* = 0.038]. The above results generally suggest that in older groups, GG homozygosity may be associated with poorer cognitive function performance. Furthermore, although GG homozygotes and BNT scores did not show significant associations in either age group, their interaction with age was still statistically significant (*p* for interaction = 0.042), suggesting a potential directional moderating effect, as shown in Figure 3A.

In the subgroup of male, GG homozygotes was significantly negatively correlated with the MMSE [β = −2.23, 95% CI (−3.53, –0.93), *p* < 0.001], while in the subgroup of female, it showed a non-significant positive trend. Meanwhile, the interaction between rs440446 and gender in the MMSE was statistically significant (*p* for interaction = 0.008). In the AVLT-N5, GG homozygotes showed a significant negative correlation in males [β = −1.18, 95% CI (−2.35, –0.01), *p* = 0.049]. Furthermore, in the SCWT-C (right), GG homozygotes were significantly negatively correlated with the score in males [β = −3.53, 95% CI (−6.32, –0.75), *p* = 0.014], while showing a non-significant positive trend in females. Moreover, the interaction between rs440446 and gender was significant in SCWT-C (right) (*p* for interaction = 0.022). In the TMT-A, GG homozygotes were significantly positively correlated with the required time in males [β = 17.56, 95% CI (4.58, 30.55), *p* = 0.009], while no significant association was observed in females. Moreover, the interaction between rs440446 and gender in this test reached a significant level (*p* for interaction = 0.048). In the female subgroup, GG homozygotes and the total score of VFT [β = −4.46, 95% CI (−7.73, –1.18), *p* = 0.008] and its three sub-items—VFT-Animal [β = −1.58, 95% CI (−3.12, –0.04), *p* = 0.046], VFT-vegetable [β = −1.66, 95% CI (−3.06, –0.27), *p* = 0.020] and VFT-fruit [β = −1.23, 95% CI (−2.43, –0.03), *p* = 0.045] all showed a significant negative correlation. It is notable that a significant negative correlation between GG homozygotes and VFT-fruit scores was also observed in male subgroup. The above results suggest that there are significant gender differences in the impact of GG homozygotes of rs440446 on cognitive function as shown in Figure 3B.

Among APOE ε4 non-carriers, GG homozygotes exhibited associations with multiple cognitive deficits compared with C allele carriers: prolonged TMT-A completion times [β = 12.41, 95% CI (2.05, 22.76), *p* = 0.019], indicating impaired visual search efficiency, worse score for VFT [β = −4.47, 95% CI (−7.71, –1.24), *p* = 0.007], VFT-animal [β = −1.96, 95% CI (−3.51, –0.41), *p* = 0.014], and VFT-fruit [β = −1.48, 95% CI (−2.63, –0.32), *p* = 0.013], which may indicate that negative effects of GG homozygotes on language function. In subgroup of APOE ε4 carriers, GG homozygotes showed no statistically significant associations with multiple cognitive tests. Interaction analyses revealed no significant interaction effects of APOE ε4 carrying status and rs440446 on cognitive performance (all *p* for interaction > 0.05) as shown in Figure 3C.

### Association between rs440446 and lipid and metabolic markers

In the subgroup analysis of lipid profiles and metabolic markers among 326 participants (C allele carriers *n* = 293, GG homozygotes *n* = 33), there were no significant differences in baseline data (Supplementary Table S1) or levels of HDL, LDL, TC, TG, HbA1c and HCY (Supplementary Table S2) except for APOE carrier proportion between C allele carriers and GG homozygous carriers. After adjusting for covariates, the four-stage linear regression model confirmed no significant association between rs440446 GG genotype, blood lipids, and metabolic markers (Supplementary Table S3). These non-significant findings suggest that rs440446 polymorphisms may have limited effects on this population’s lipid homeostasis or glucose metabolism. In the sensitivity analysis of rs440446 and multi-dimensional cognitive function using lipid and metabolic subgroup data, rs440446 GG homozygosity remained significantly associated with poor language function (Supplementary Tables S4, S5).

### Propensity score matching for rs-fMRI subgroup

A notable imbalance in sample size was observed between C allele carriers (*n* = 196) and GG homozygotes (*n* = 20) in the rs-fMRI subgroup, which was addressed by performing 1:1 nearest-neighbor PSM based on sex, age, education, and BMI to balance the unequal group sizes. After matching, 40 participants (20 in each group) were retained, as shown in Table 4. Before PSM, the GG homozygotes exhibited significantly lower educational years compared to the C allele carriers (9.60 ± 2.91 vs. 11.23 ± 3.51, *p* = 0.047) and had a notably higher APOE ε4 carrier rate (55.00 vs. 12.24%, *p* < 0.001). Cognitive assessments revealed that the GG homozygous group performed significantly worse on several cognitive measures: AVLT (N4, 3.90 ± 3.77 vs. 5.28 ± 2.77, *p* = 0.042; N5, 3.35 ± 3.62 vs. 4.89 ± 2.82, *p* = 0.025), ROCF (recall, 8.80 ± 6.46 vs. 13.68 ± 8.26, *p* = 0.011), SDMT (28.13 ± 9.90 vs. 35.75 ± 13.17, *p* = 0.013), TMT-A (76.50 ± 47.91 vs. 59.27 ± 23.92 s, *p* = 0.007), TMT-B (182.20 ± 62.89 vs. 155.07 ± 57.61 s, *p* = 0.048), VFT (36.05 ± 11.26 vs. 41.79 ± 9.75, *p* = 0.014), and its subcomponents (animal: 13.85 ± 4.93 vs. 16.19 ± 4.41, *p* = 0.026; vegetable: 11.60 ± 4.88 vs. 13.80 ± 3.95, *p* = 0.022).

**TABLE 4:** Baseline characteristics before and after propensity score matching in the rs-fMRI subgroup.

	Before			After		
Variables	CC, CG (*n* = 196)	GG (*n* = 20)	*p*-value	CC, CG (*n* = 20)	GG (*n* = 20)	*p*-value
Age (years)	61.90 ± 8.25	62.35 ± 8.36	0.816	63.85 ± 6.93	62.35 ± 8.36	0.540
Education (years)	11.23 ± 3.51	9.60 ± 2.91	0.047*	10.25 ± 3.11	9.60 ± 2.91	0.499
BMI	24.21 ± 2.85	24.36 ± 3.49	0.834	23.87 ± 2.57	24.36 ± 3.49	0.619
Gender			0.522			0.256
Male	73 (37.24%)	6 (30.00%)		17 (85.00%)	14 (70.00%)	
Female	123 (62.76%)	14 (70.00%)		3 (15.00%)	6 (30.00%)	
APOE ε 4 carriers			<0.001***			0.008**
No	172 (87.76%)	9 (45.00%)		17 (85.00%)	9 (45.00%)	
Yes	24 (12.24%)	11 (55.00%)		3 (15.00%)	11 (55.00%)	
Hypertension			0.774			0.736
No	121 (61.73%)	13 (65.00%)		14 (70.00%)	13 (65.00%)	
Yes	75 (38.27%)	7 (35.00%)		6 (30.00%)	7 (35.00%)	
Diabetes			0.538			0.376
No	167 (85.20%)	16 (80.0%)		18 (90.00%)	16 (80.00%)	
Yes	29 (14.8%)	4 (20.0%)		2 (10.00%)	4 (20.00%)	
Cerebrovascular disease			0.178			0.705
No	169 (86.22%)	15 (75.00%)		16 (80.00%)	15 (75.00%)	
Yes	27 (13.78%)	5 (25.00%)		4 (20.00%)	5 (25.00%)	
Cardiovascular disease			0.791			0.633
No	162 (82.65%)	17 (85.00%)		18 (90.00%)	17 (85.00%)	
Yes	34 (17.35%)	3 (15.00%)		2 (10.00%)	3 (15.00%)	
Hyperlipemia			0.739			0.507
No	144 (73.46%)	14 (70.00%)		12 (60.00%)	14 (70.00%)	
Yes	52 (26.54%)	6 (30.00%)		8 (40.00%)	6 (30.00%)	
Smoking			0.802			0.151
No	152 (77.55%)	16 (80.00%)		19 (95.00%)	16 (80.00%)	
Yes	44 (22.45%)	4 (20.00%)		1 (5.00%)	4 (20.00%)	
Alcohol use			0.690			1.000
No	149 (76.02%)	16 (80.00%)		16 (80.00%)	16 (80.00%)	
Yes	47 (24.98%)	4 (20.00%)		4 (20.00%)	4 (20.00%)	

Continuous variables are presented as mean ± standard deviation (SD), while categorical variables are expressed as frequency (percentage). Intergroup differences between the rs440446 C allele carrier group and GG homozygous group were assessed using independent two-sample *t*-tests for continuous variables and Chi-square (χ^2^) tests for categorical variables. Statistical significance was defined as a two-tailed *p* < 0.05. BMI, body mass index; APOE, Apolipoprotein E. *p < 0.05; **p < 0.01; ***p < 0.001.

Following PSM adjustment, while most demographic and comorbidity indicators showed no significant differences between the groups (all *p* > 0.05), the discrepancy in APOE ε4 carrier rates persisted (GG homozygous group 55.00% vs. C allele carriers group 15.00%, *p* = 0.008). Despite baseline characteristics becoming more balanced, the GG homozygous group still demonstrated cognitive disadvantages post-matching: AVLT (N5, 3.35 ± 3.62 vs. 5.45 ± 2.86, *p* = 0.049) and SDMT (28.13 ± 9.90 vs. 35.30 ± 10.14, *p* = 0.029). Notably, after matching, differences across other cognitive tests, such as TMT-A/B and VFT subcomponents, were no longer significant (all *p* > 0.05), as shown in Table 5.

**TABLE 5:** Multidimensional cognitive function score before and after propensity score matching in the rs-fMRI subgroup.

Variables	Before			After		
	CC, CG (*n* = 196)	GG (*n* = 20)	*p*-value	CC, CG (*n* = 20)	GG (*n* = 20)	*p*-value
MMSE	27.01 ± 2.83	24.90 ± 4.45	0.050	26.70 ± 4.03	24.90 ± 4.45	0.188
AVLT (N1–N3)	16.57 ± 5.56	15.00 ± 7.23	0.246	16.40 ± 5.88	15.00 ± 7.23	0.506
AVLT (N4)	5.28 ± 2.77	3.90 ± 3.77	0.042*	5.35 ± 2.78	3.90 ± 3.77	0.174
AVLT (N5)	4.89 ± 2.82	3.35 ± 3.62	0.025*	5.45 ± 2.86	3.35 ± 3.62	0.049*
AVLT (total)	26.74 ± 10.47	22.20 ± 14.19	0.076	27.25 ± 11.03	22.20 ± 14.19	0.217
ROCF (copy)	32.61 ± 6.77	31.10 ± 7.45	0.348	32.50 ± 8.08	31.10 ± 7.45	0.572
ROCF (recall)	13.68 ± 8.26	8.80 ± 6.46	0.011*	11.50 ± 7.76	8.80 ± 6.46	0.239
CDT	22.06 ± 5.62	21.25 ± 8.37	0.560	23.30 ± 5.14	21.25 ± 8.37	0.356
SCWT-A (time)	28.77 ± 8.06	32.05 ± 11.22	0.097	29.05 ± 12.22	32.05 ± 11.22	0.424
SCWT-A (right)	49.65 ± 1.61	49.80 ± 0.89	0.678	43.15 ± 21.42	42.15 ± 11.35	0.840
SCWT-B (time)	41.95 ± 14.88	42.15 ± 11.35	0.954	43.15 ± 21.42	42.15 ± 11.35	0.855
SCWT-B (right)	49.35 ± 1.50	48.80 ± 1.94	0.129	49.60 ± 0.75	48.80 ± 1.94	0.093
SCWT-C (time)	89.29 ± 32.91	93.30 ± 43.13	0.615	90.65 ± 36.31	93.30 ± 43.13	0.835
SCWT_C (right)	46.27 ± 5.16	43.85 ± 8.60	0.065	46.50 ± 6.93	43.85 ± 8.60	0.290
SDMT	35.75 ± 13.17	28.13 ± 9.90	0.013*	35.30 ± 10.14	28.13 ± 9.90	0.029*
TMT-A	59.27 ± 23.92	76.50 ± 47.91	0.007**	61.30 ± 28.61	76.50 ± 47.91	0.231
TMT-B	155.07 ± 57.61	182.20 ± 62.89	0.048*	151.20 ± 50.52	182.20 ± 62.89	0.094
VFT	41.79 ± 9.75	36.05 ± 11.26	0.014*	40.05 ± 9.94	36.05 ± 11.26	0.241
VFT-animal	16.19 ± 4.41	13.85 ± 4.93	0.026*	15.15 ± 4.03	13.85 ± 4.93	0.367
VFT-vegetable	13.80 ± 3.95	11.60 ± 4.88	0.022*	13.25 ± 3.80	11.60 ± 4.88	0.240
VFT-fruit	11.91 ± 3.43	10.60 ± 3.35	0.105	11.65 ± 3.13	10.60 ± 3.35	0.312
BNT	22.9 ± 3.8	20.2 ± 4.8	0.004	21.70 ± 4.08	20.20 ± 4.82	0.295

MMSE, Mini-Mental State Examination; AVLT, Auditory Verbal Learning Test; ROCF, Rey-Osterrieth Complex Figure; CDT, Clock Drawing Test; SCWT, Stroop Color Word Test; SDMT, Symbol Digit Modalities Test; TMT, Trail Making Test; VFT, Verbal Fluency Test; BNT: Boston Naming. *p < 0.05; **p < 0.01.

### Differences in fALFF between GG homozygous and C allele carriers

**FIGURE 4:**
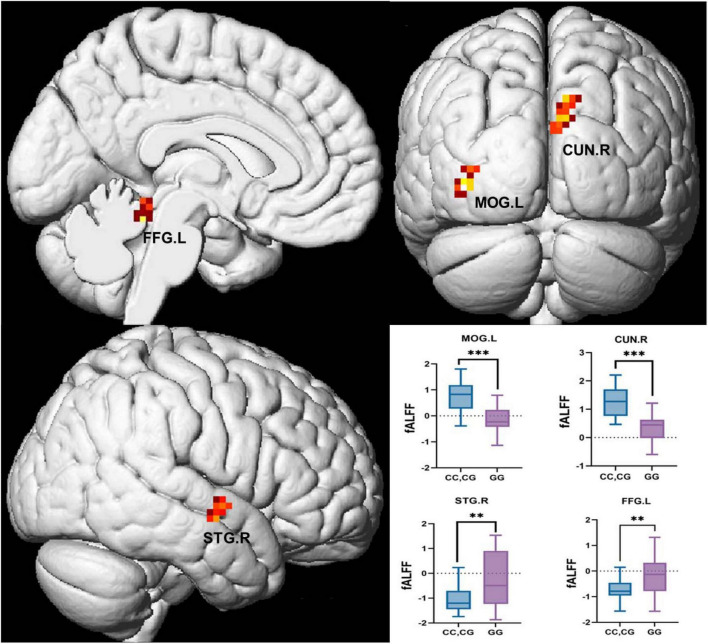
Brain regions with significant differences in fALFF analysis between GG homozygotes and C allele carriers. BA, Brodmann Area; MOG.L, Left middle occipital gyrus; CUN.R, Right cuneus; STG.R, Right superior temporal gyrus; FFG.L, Left fusiform gyrus. All clusters are corrected by Cluster-wise FDR (voxel-wise *p* < 0.001 and cluster-wise *p* < 0.05).

GLMs were utilized to analyze differences in fALFF between the two groups after PSM. C allele carriers exhibited significantly higher fALFF in the MOG.L and CUN.R compared with GG homozygotes. In contrast, fALFF in the STG.R and FFG.L was significantly higher in the GG homozygous group than in the C allele carriers (Figure 4). All results were corrected for multiple comparisons using cluster-level FDR at the cluster level (single voxel *p* < 0.001), as detailed in Table 6.

**TABLE 6:** The differential brain regions between C allele carriers and GG homozygotes after fALFF analysis.

Brain regions (BA)	Cluster size	*X*	*Y*	*Z*	Peak value
MOG.L (19)	24	−39	−84	−3	5.2065
CUN.R (18)	37	6	−75	27	4.2404
STG.R (48)	36	48	−3	−3	−5.2768
FFG.L (30)	25	−21	−42	−12	−5.3331

BA, Brodmann Area; MOG.L, Left middle occipital gyrus; CUN.R, Right cuneus; STG.R, Right superior temporal gyrus; FFG.L, Left fusiform gyrus. All clusters are corrected by Cluster-level FDR (voxel-wise *p* < 0.001 and cluster-wise *p* < 0.05).

**FIGURE 5:**
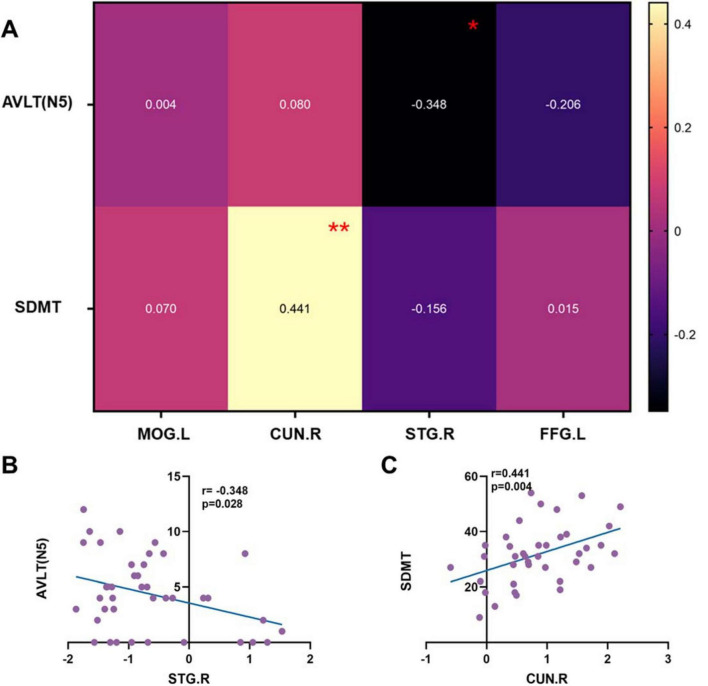
The correlations of ROIs and neuropsychological tests. **(A)** Heat maps of correlation coefficients for all ROIs and AVLT (N5) and SDMT. **(B)** Scatter plot of the correlation between STG.R and AVLT (N5). **(C)** Scatter plot of the correlation between CUN.R and SDMT. AVLT, Auditory Verbal Learning Test; SDMT, Symbol Digit Modalities Test; CUN.R, Right cuneus; STG.R, Right superior temporal gyrus; *r*, correlation coefficient. *p < 0.05; **p < 0.01.

The above differential brain regions were defined as ROIs. After PSM, neuropsychological assessment showed that the two groups differed only in AVLT (N5) and SDMT. Pearson correlations between the fALFF values of the ROIs and AVLT (N5) as well as SDMT were therefore examined (Figure 5A). STG.R was negatively correlated with the AVLT-N5 (*r* = −0.348, *p* = 0.028, Figure 5B), indicating that higher neural activity in the STG.R is associated with poorer long-delayed recall performance. Combined with the previous results (Figure 4), although the GG homozygous group had higher neural activity in STG.R, it was instead associated with poorer performance of AVLT (N5). The CUN.R showed a significant positive correlation with SDMT (*r* = 0.441, *p* = 0.004, Figure 5C), indicating that increased neural activity in the CUN.R is associated with better SDMT performance, which may explain why C allele carriers exhibited higher SDMT scores.

## Discussion

The baseline characteristics showed no significant differences between GG homozygotes and C allele carriers. However, the GG homozygotes exhibited a higher proportion of APOE ε4 carriers. It demonstrated poorer performance in neuropsychological tests of general cognitive function, memory, visual search ability, attention, and language function. To account for confounding effects from covariates, including APOE ε4, we incorporated significantly differing scales into multivariate linear regression analyses adjusted across four models (both Model 3 and Model 4 include APOE ε4). After these adjustments, GG homozygotes remained negatively associated with VFT and VFT-fruit scores. This suggests that the rs440446 GG genotype may have a more pronounced impact on language function than on other cognitive domains. Subsequent subgroup analyses showed that in the subgroup of ≥ 60 years, the female subgroup, and the APOE ε4 non-carrier subgroup, GG homozygotes were significantly associated with poorer language function. Previous studies have reported APOE promoter SNPs (e.g., rs405509, rs449647) associated with AD risk independent of APOE ε4 status (Bizzarro et al., 2009; Wang et al., 2000). Notably, although current genetic studies in elderly Chinese populations have not revealed associations between APOE intron region polymorphisms and AD risk, this study first reports that the GG genotype at the rs440446 locus within this region is associated with poorer multidimensional cognitive performance, especially in terms of worse language functions. A cross-sectional study integrating multiple databases in the European population found that after completely excluding APOE ε4 carriers, the rs440446 C allele enhanced the beneficial effect of APOE ε2 on AD in a male population. In contrast, GG homozygotes exhibited no such effect (Kulminski et al., 2021). Although longitudinal data suggested divergent trajectories for visuospatial ability in male populations (rs440446 C allele carriers showing age-related improvement *vs*. GG homozygotes declining) (Rantalainen et al., 2016), this study detected no group differences in visuospatial performance using ROCF-copy and CDT. This may indicate either that GG homozygotes may preferentially impair language function in elderly Chinese individuals or that the observed differences are due to research data and method variations. However, these studies collectively reveal that GG homozygotes damage different cognitive domains.

Additionally, although the rs440446 is located within an intron of the APOE gene and is not involved in coding the mature mRNA, we still investigated its association with blood lipids and metabolic markers. No significant differences and associations were observed in this study for LDL, HDL, TC, TG, HbA1c, and HCY between GG homozygotes and C allele carriers. These null findings suggest that the functional impact of rs440446 on systemic lipid metabolism may be limited in this population or may depend on specific environmental or genetic contexts. Notably, a cross-sectional study conducted in a Shanghai Chinese cohort reported that among individuals with high red meat consumption, G allele carriers of rs440446 exhibited significantly lower TC and LDL levels compared to CC homozygotes under similar dietary conditions (Andreotti et al., 2009). While these findings imply a possible gene-diet interaction, the underlying mechanisms remain poorly understood. Another nested case-control study found that the fetal rs440446 G allele was associated with increased risk of maternal supraphysiological hypercholesterolemia (MSPH) (Cai et al., 2022), suggesting a potential role of this variant in lipid homeostasis during pregnancy, though its generalizability to non-pregnant populations remains uncertain. Taken together, these mixed results highlight the complexity of rs440446’s influence on lipid metabolism. Our findings do not support a significant effect of rs440446 on lipid markers in the studied cohort; however, they do not rule out context-dependent effects, such as those modulated by diet, hormonal status, or developmental stage. Given the increasing interest in non-coding variants and their regulatory roles, further investigations—particularly longitudinal studies incorporating gene-environment interactions—are warranted to clarify the functional significance of rs440446 and other intronic SNPs within the APOE locus.

In the rs-fMRI subgroup analysis, after PSM, GG homozygotes still performed worse than C allele carriers on AVLT(N5) and SDMT. In the subsequent fALFF analysis, decreased spontaneous activity was observed in the MOG.L and CUN.R regions of GG homozygotes, while increased spontaneous activity was found in the STG.R and FFG.L regions. After the correlation analysis between AVLT (N5), SDMT, and ROIs was established, we found that STG.R was negatively correlated with AVLT (N5). STG.R is considered to be an important node in the cortical-hippocampus network involved in the regulation of long-delayed recall (Ma et al., 2023), and poor spontaneous neural activity of STG.R in GG homozygous carriers may be the reason for worse long-delayed recall in them. CUN.R negatively correlated with the SDMT. Cuneus is vital in transsaccadic processing (Baltaretu et al., 2021), which is a key part of visual information processing. In this study, the vertex coordinates of CUN.R were located in Brodmann Area 18, an important part of the secondary visual cortex (V2) (Leopold, 2012). Decreased CUN.R activity may directly impair the shunt efficiency of visual information(Freud et al., 2017), which is highly consistent with the decreased SDMT performance in the GG homozygous group. SDMT is primarily used to assess information processing and attention, but the actual process requires the subject to observe the consistency between numbers and symbols constantly, which relies on coordinating the dorsal pathway’s spatial coding and the ventral pathway’s symbol recognition (Freud et al., 2017; Silva et al., 2018). rs440446 GG genotype may cause a decrease in the speed of vision-motor conversion by impacting V2’s ability to preprocess information (e.g., by reducing neural synchronization). This mechanism is partially similar to the pathological process of visual hallucination caused by cuneus atrophy in Lewy body dementia (Delli Pizzi et al., 2014; Woyk et al., 2023). Previous longitudinal data have shown that rs440446 has a differentiated effect on visuospatial ability (Rantalainen et al., 2016), and the decreased spontaneous neural activity in the CUN.R of the secondary visual cortex caused by GG homozygote may be one of the reasons. Although we found no inter-group differences between ROCF-copy and CDT in assessing visuospatial ability, there were significant differences in SDMT. This suggests that rs440446 may interfere with visual information processing rather than isolated visuospatial ability or that visuospatial decline in the sample may not have reached the scale’s detection threshold.

This study integrated multidimensional cognitive function data, lipid metabolism indicators, and neuroimaging evidence to systematically investigate the impact of the APOE rs440446 polymorphism on cognition and brain function in Chinese urban community-dwelling older adults. Nevertheless, certain limitations should be acknowledged. In the neuroimaging analysis, the sample size became relatively small after PSM, which may compromise the stability of the observed neural activity differences and limit the generalizability of the findings. Additionally, there was a significant difference in the proportion of APOE ε4 carriers between the two groups. Although we controlled for this variable as a covariate in the fALFF analysis, its potential confounding effect cannot be entirely ruled out. These preliminary findings, however, offer a foundation for further exploration into the functional significance of rs440446, particularly in relation to language processing and spontaneous brain activity.

## Conclusion

Taken together, this cross-sectional study demonstrated significant differences in language function between rs440446 GG homozygotes and C allele carriers, the association between GG homozygosity and poorer language performance was consistently observed in key subgroups including individuals ≥ 60 years of age, females, and APOE ε4 non-carriers. However, the rs-fMRI subgroup analysis did not identify specific language-associated brain regions, suggesting a need for more sensitive neuroimaging approaches or the inclusion of more subjects. As the first comprehensive investigation into the effects of rs440446 on multidimensional cognition, metabolic profiles, and resting-state neural activity in the Chinese urban elderly population, our findings suggest that this intronic variant may play a subtle but meaningful role in cognitive aging. Future studies integrating longitudinal neuroimaging with cellular or animal models are warranted to elucidate the regulatory mechanisms of APOE intronic variants such as rs440446. Such integrative approaches will not only deepen our understanding of AD pathogenesis but also inform the development of targeted therapeutic and preventive strategies.

## Data Availability

The authors will make the raw data supporting this article’s conclusions available without undue reservation.

## Footnotes

1
http://www.restfmri.net/forum/RESTplus

2
https://www.fil.ion.ucl.ac.uk/spm/software/spm12/

3
www.nitrc.org/projects/dcm2nii/

4
www.zstats.net

5
https://www.ibm.com/cn-zh/spss
