# Supplementary material for: Association of rs440446 in the APOE intron region with multidimensional cognitive function, lipid/metabolic markers, and spontaneous neural activity in Chinese urban community-dwelling older adults
Source: Front Neurosci. 2025 Jul 16;19:1629254. doi: 10.3389/fnins.2025.1629254 (PMC12307423; doi:10.3389/fnins.2025.1629254)
Supplement: Supplementary file 1 [file Table_1.docx]

| Variables | CC, CG  (n=293) | GG  (n=33) | Statistical | p-value |
| --- | --- | --- | --- | --- |
| Age (years) | 61.70 ± 8.60 | 63.18 ± 9.03 | t=-0.93 | 0.352 |
| Education (years) | 10.88 ± 3.61 | 10.77 ± 3.91 | t = 0.15 | 0.878 |
| BMI | 24.45 ± 3.89 | 23.79 ± 3.80 | t = 0.93 | 0.351 |
| Gender, n(%) |  |  | χ²=0.08 | 0.775 |
| male | 123 (41.98%) | 13 (39.39%) |  |  |
| female | 170 (58.02%) | 20 (60.61%) |  |  |
| APOE ε4 carrier, n(%) |  |  | χ²=12.22 | <0.001*** |
| no | 249 (84.98%) | 20 (60.61%) |  |  |
| yes | 44 (15.02%) | 13 (39.39%) |  |  |
| Hypertension, n(%) |  |  | χ²=1.71 | 0.191 |
| no | 179 (61.09%) | 24 (72.73%) |  |  |
| yes | 114 (38.91%) | 9 (27.27%) |  |  |
| Diabetes, n(%) |  |  | χ²=0.11 | 0.554 |
| no | 251 (85.67%) | 27 (81.81%) |  |  |
| yes | 42 (14.33%) | 6 (18.19%) |  |  |
| Cerebrovascular disease, n(%) |  |  | χ²=0.05 | 0.825 |
| no | 235 (80.20%) | 27 (81.81%) |  |  |
| yes | 58 (19.80%) | 6 (18.19%) |  |  |
| Cardiovascular disease, n(%) |  |  | χ²=1.72 | 0.190 |
| no | 248 (84.64%) | 25 (75.76%) |  |  |
| yes | 45 (15.36%) | 8 (24.24%) |  |  |
| Hyperlipemia, n(%) |  |  | χ²=0.33 | 0.565 |
| no | 208 (70.99%) | 25 (75.76%) |  |  |
| yes | 85 (29.01%) | 8 (24.24%) |  |  |
| Smoking, n(%) |  |  | χ²=2.34 | 0.126 |
| no | 223 (76.11%) | 29 (87.88%) |  |  |
| yes | 70 (23.89%) | 4 (12.12%) |  |  |
| Alcohol use, n(%) |  |  | χ²=0.26 | 0.610 |
| no | 219 (74.74%) | 26 (78.79%) |  |  |
| yes | 74 (25.3%) | 7 (21.21%) |  |  |

Supplementary material

Table S1. Baseline data of C allele carriers and GG homozygotes in the lipid and metabolic markers subgroup

Continuous variables are presented as mean ± standard deviation (SD), while categorical variables are expressed as frequency (percentage). Intergroup differences between the rs440446 C allele carrier group and GG homozygous group were assessed using independent two-sample t-tests for continuous variables and Chi-square (χ²) tests for categorical variables. Statistical significance was defined as a two-tailed p < 0.05. BMI: body mass index; APOE: Apolipoprotein E. *: p<0.05; **:p<0.01; ***: p<0.001.

Table S2. Comparison of blood lipid and metabolic markers between C allele carriers and GG homozygotes

|  | Variables | CC, CG  (n=293) | GG  (n=33) | Statistical | p-value |
| --- | --- | --- | --- | --- | --- |
| Blood Lipid | HDL | 1.38 ± 0.73 | 1.42 ± 0.34 | t = -0.32 | 0.747 |
|  | LDL | 3.01 ± 0.81 | 2.96 ± 0.79 | t = 0.35 | 0.727 |
|  | TC | 5.13 ± 1.14 | 5.31 ± 1.17 | t = -0.83 | 0.386 |
|  | TG | 2.09 ± 1.37 | 2.18 ± 1.56 | t = -0.35 | 0.726 |
| Metabolic markers | HBA1C | 5.74 ± 2.12 | 5.77 ± 1.93 | t = -0.06 | 0.950 |
|  | HCY | 11.89 ± 5.89 | 12.46 ± 6.59 | t = -0.52 | 0.601 |

|  | Variables | Reference | Model 1 | Model 2 | | Model 3 | | | Model 4 |
| --- | --- | --- | --- | --- | --- | --- | --- | --- | --- |
|  |  |  | β(95% CI)  p-value | β(95% CI)  p-value | | β(95% CI)  p-value | | | β(95% CI)  p-value |
| Blood lipid | HDL | CC, CG | 0.04 (-0.21, 0.30) 0.747 | | 0.04 (-0.21, 0.29) 0.756 | | 0.05 (-0.20, 0.30) 0.704 | 0.06 (-0.19, 0.32) 0.618 | |
|  | LDL | CC, CG | -0.05 (-0.34, 0.24) 0.754 | | -0.05 (-0.34, 0.24) 0.744 | | -0.11 (-0.41, 0.18)  0.440 | -0.13 (-0.42, 0.16) 0.382 | |
|  | TC | CC, CG | 0.17 (-0.24, 0.59)  0.407 | | 0.17 (-0.24, 0.58) 0.410 | | 0.11 (-0.31, 0.52) 0.611 | 0.09 (-0.31, 0.50) 0.655 | |
|  | TG | CC, CG | 0.09 (-0.41, 0.59)  0.726 | | 0.12 (-0.38, 0.61) 0.640 | | 0.10 (-0.39, 0.59) 0.693 | 0.06 (-0.43, 0.56) 0.800 | |
| Metabolic markers | HBA1C | CC, CG | 0.02 (-0.73, 0.78) 0.950 | | 0.01 (-0.75, 0.77) 0.985 | | 0.01 (-0.77, 0.79) 0.981 | 0.02 (-0.76, 0.80) 0.953 | |
|  | HCY | CC, CG | 0.57 (-1.57, 2.72) 0.601 | | 0.44 (-1.57, 2.46) 0.668 | | 0.10 (-1.95, 2.16) 0.922 | -0.13 (-2.19, 1.94) 0.905 | |

HDL: High-Density Lipoprotein; LDL: Low-Density Lipoprotein; TC: Total Cholesterol; TG: Triglycerides. HbA1c: Hemoglobin A1c; HCY: Homocysteine. Continuous variables are presented as mean ± standard deviation (SD); Intergroup differences between the rs440446 C allele carrier group and GG homozygous group were assessed using independent two-sample t-tests for continuous variables.

Table S3. Correlation of rs440446(CC, CG vs. GG) with blood lipids and metabolic markers.

HDL: High-Density Lipoprotein; LDL: Low-Density Lipoprotein; TC: Total Cholesterol; TG: Triglycerides. HbA1c: Hemoglobin A1c; HCY: Homocysteine. Model 1: crude. Model 2: adjust gender, age, and education. Model3: Model 2+ BMI+APOE ε4. Model 4: Model 3 + hypertension, diabetes (exclude in HBA1C analysis), cerebrovascular disease, cardiovascular disease, hyperlipemia (exclude in blood lipid analysis), smoking, drinking.

| Variables | CC, CG  (n=293) | GG  (n=33) | Statistic | p-value |
| --- | --- | --- | --- | --- |
|  |  |  |  |  |
| MMSE | 26.79 ± 2.98 | 26.03 ± 4.22 | t=1.00 | 0.322 |
| AVLT(N1-N3) | 15.59 ± 5.41 | 15.15 ± 6.58 | t=0.43 | 0.666 |
| AVLT(N4) | 4.95 ± 2.72 | 4.94 ± 3.18 | t=0.01 | 0.991 |
| AVLT(N5) | 4.54 ± 2.81 | 4.36 ± 3.24 | t=0.34 | 0.733 |
| AVLT(total) | 25.06 ± 10.31 | 24.42 ± 12.36 | t=0.33 | 0.742 |
| ROCF(copy) | 31.61 ± 7.85 | 32.06 ± 8.09 | t=-0.31 | 0.754 |
| ROCF(recall) | 13.32 ± 8.41 | 12.24 ± 7.22 | t=0.71 | 0.478 |
| CDT | 21.81 ± 6.18 | 22.48 ± 6.67 | t=-0.59 | 0.555 |
| SCWT-A(time) | 29.47 ± 10.07 | 30.61 ± 8.76 | t=-0.62 | 0.536 |
| SCWT-A(right) | 49.62 ± 1.64 | 49.85 ± 0.36 | t=-0.78 | 0.436 |
| SCWT-B(time) | 42.85 ± 16.60 | 47.73 ± 26.98 | t=-1.49 | 0.138 |
| SCWT-B(right) | 49.00 ± 3.18 | 48.97 ± 2.20 | t=0.06 | 0.953 |
| SCWT-C(time) | 93.54 ± 33.73 | 99.82 ± 37.98 | t=-1.00 | 0.317 |
| SCWT-C(right) | 45.35 ± 6.10 | 45.45 ± 5.00 | t=-0.10 | 0.923 |
| SDMT | 34.03 ± 13.88 | 30.62 ± 12.81 | t=1.35 | 0.179 |
| TMT-A | 61.78 ± 29.13 | 68.64 ± 30.67 | t=-1.27 | 0.204 |
| TMT-B | 165.16 ± 72.90 | 164.30 ± 67.46 | t=0.06 | 0.949 |
| VFT | 40.96 ± 9.51 | 36.61 ± 10.40 | t=2.47 | 0.014* |
| VFT-animal | 15.69 ± 4.09 | 14.27 ± 3.87 | t=1.89 | 0.059 |
| VFT-vegetable | 13.37 ± 3.94 | 11.76 ± 4.74 | t=2.18 | 0.030* |
| VFT-Fruit | 11.94 ± 3.33 | 10.58 ± 3.55 | t=2.22 | 0.027* |
| BNT | 22.59 ± 4.15 | 22.73 ± 4.24 | t=-0.18 | 0.855 |

Table S4. Multidimensional cognitive function differences between C allele carriers and GG homozygotes in blood lipid and metabolic markers subgroup

MMSE: Mini-Mental State Examination; AVLT: Auditory Verbal Learning Test; ROCF: Rey-Osterrieth Complex Figure; CDT: Clock Drawing Test; SCWT: Stroop Color Word Test; SDMT: Symbol Digit Modalities Test; TMT: Trail Making Test; VFT: Verbal Fluency Test. BNT: Boston Naming Test. *: p<0.05; **:p<0.01; ***: p<0.001. Independent two-sample t-tests obtained the p-values.

Table S5. Correlation of rs440446(CC, CG vs. GG) with neuropsychological tests.

| Variables | Reference | Model1 | Model2 | Model3 | Model4 |
| --- | --- | --- | --- | --- | --- |
|  |  | β (95%CI)  p-value | β (95%CI)  p-value | β (95%CI)  p-value | β (95%CI)  p-value |
| VFT | CC, CG | -4.36 (-7.81, -0.90)  0.014* | -3.85 (-6.95, -0.75)  0.015* | -3.47 (-6.63, -0.30)  0.033* | -3.38 (-6.48, -0.29)  0.033* |
| VFT-vegetable | CC, CG | -1.61 (-3.06, -0.17)  0.030* | -1.40 (-2.70, -0.11)  0.035* | -1.33 (-2.65, -0.00)  0.050 | -1.26 (-2.58, 0.06)  0.062 |
| VFT-fruit | CC, CG | -1.37 (-2.57, -0.16)  0.027* | -1.27 (-2.40, -0.13)  0.029* | -1.14 (-2.29, 0.02)  0.055 | -1.18 (-2.30, -0.05)  0.042* |

VFT: Verbal Fluency Test. Model 1: crude. Model2: adjust: gender, age, education. Model3: Model 2 + BMI, APOE ε4. Model4: Model 3 + hypertension, diabetes, cerebrovascular disease, cardiovascular disease, hyperlipemia, smoking, drinking.
